# First Middle East Experience with Novel Foam Dressing Together with Negative Pressure Wound Therapy and Instillation

**DOI:** 10.7759/cureus.3415

**Published:** 2018-10-05

**Authors:** Muneera E Ben-Nakhi, Hazem I Eltayeb

**Affiliations:** 1 Plastic Surgery, Adan Hospital, Ahmadi, KWT; 2 Plastic Surgery, Adan Hospital, Ahmadi , KWT

**Keywords:** negative pressure wound therapy, instillation, wound cleansing, cleanse choice™ dressing

## Abstract

We describe our early experience with a novel foam dressing together with negative pressure wound therapy and instillation (NPWTi-d) for cleansing and removal of infectious materials. This is a prospective review of the clinical outcomes of three patients using V.A.C. VERAFLO™ Therapy with the V.A.C. VERAFLO CLEANSE CHOICE™ Dressing. This novel foam dressing has been designed in a unique way to help facilitate the removal of infectious material, thick fibrinous exudate, slough, and other wound bioburden.

To our best knowledge, this is the first reported case series using this dressing in the Middle East. Our preliminary results suggest that adjunctive use of NPWTi-d using V.A.C. VERAFLO CLEANSE CHOICE™ Dressing can clean large or complex wounds when complete surgical debridement is not possible or when areas of non-viable tissue remain present on the wound bed after surgical debridement.

## Introduction

Chronic wounds caused by pressure, venous stasis, or diabetes mellitus (DM) are called silent epidemics. These epidemics result in significant morbidity and increased health care costs. In the United States chronic wound affects 6.5 million people with an estimated cost of 6–15 billion dollars annually [1]. Many research studies are designed to innovate advanced wound dressings, shorten the duration of wound healing and avoid the cost of hospitalization with recurrent visits to the operating rooms (OR). Possibly, one of the biggest innovations in the 1980s was the development of negative pressure wound therapy (NPWT) using the vacuum-assisted closure system (V.A.C.®Therapy, KCI, an Acelity Company, San Antonio, TX). Argenta and Morykwas [2] demonstrated that V.A.C. decreases chronic edema and promotes granulation in acute, subacute, and chronic wounds [3].

In 2002, KCI marketed the first generation of negative pressure wound therapy with instillation and dwell time (NPWTi; the V.A.C.Instill® Wound Therapy, KCI, an Acelity company, San Antonio, TX) [4]. In 2011, a more sophisticated version of the V.A.C.Instill® Wound Therapy device was introduced. This integrated second generation system is known as the V.A.C. ULTA^™ ^Therapy System with V.A.C. VERAFLO™ Instillation Therapy (KCI, an Acelity company, San Antonio, TX) [5]. Two years following its introduction, a panel of experts published international consensus guidelines designed to address the appropriate use of NPWT with intermittent instillation [6]. This consensus was followed by several published studies describing the successful use of NPWTi-d in open fractures, breast reconstruction, necrotizing fasciitis, pressure ulcers, leg ulcers, diabetic foot ulcers and other complex wounds requiring surgical debridement [7].

On March 31, 2017, V.A.C. VERAFLO CLEANSE CHOICE™ Dressing was launched in the USA, Canada, Europe and Latin America by KCI. The dressing was not distributed in our region till August 2018. This novel foam dressing consists of a unique contact layer foam with 1-cm diameter holes spaced 0.5 cm apart and two different foam thicknesses (one 0.8-cm foam and 1.6-cm thickness foam without holes) to address different wound depths. We present our first three patients who received V.A.C. VERAFLO™ Therapy using V.A.C. VERAFLO CLEANSE CHOICE™ Dressings. This small case series introduces our initial clinical experience with the new foam dressing. It is not only the first in Kuwait, but also the first reported case in the Middle East.

## Case presentation

Case 1 - infected diabetic foot

**Figure 1:**
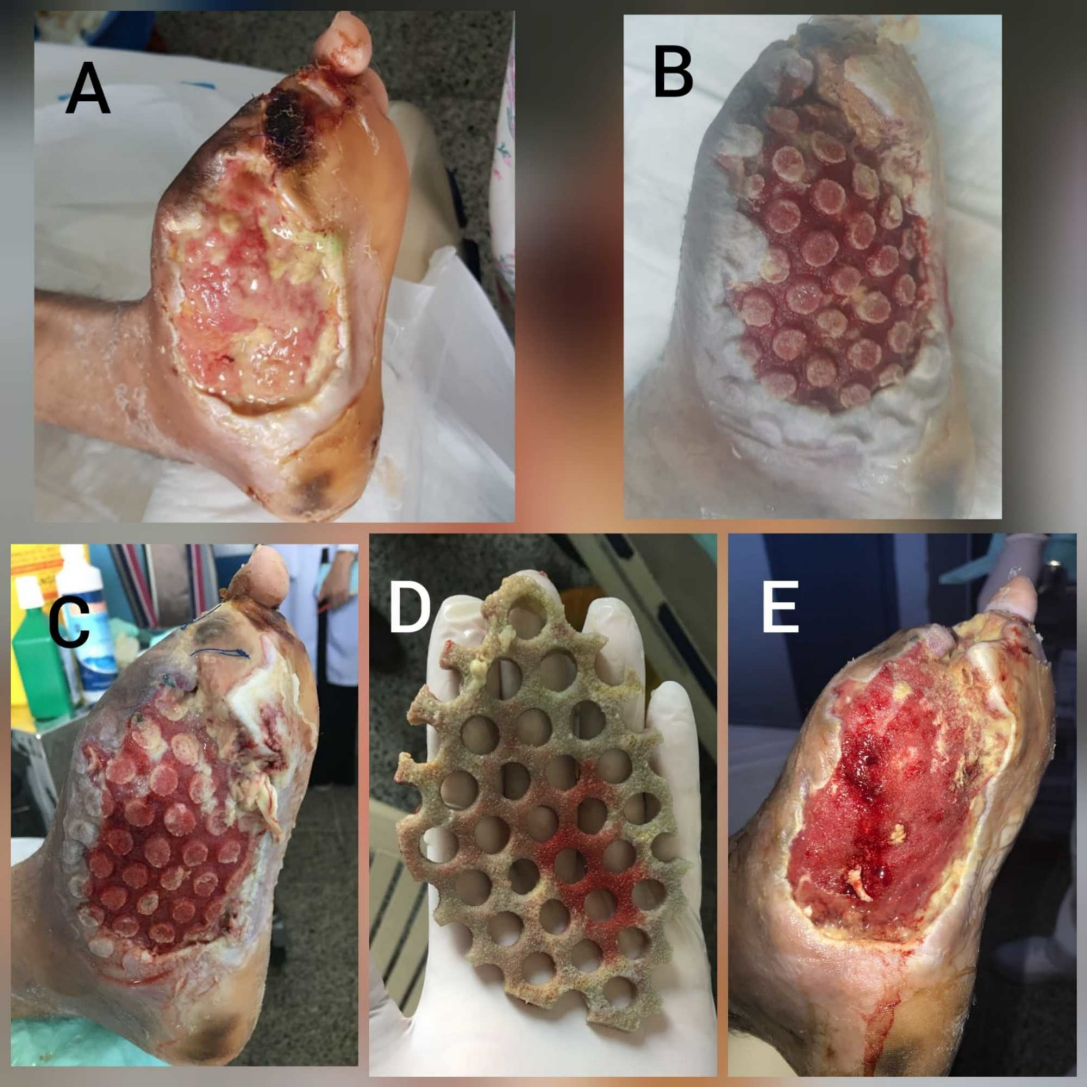
Case 1 (infected diabetic foot). (A) Left diabetic foot ulcer before dressing application. (B) After first application, the wound bed showed dramatic improvement. (C) Wound after second dressing. (D) The novel foam dressing after removal with adherent necrotic slough material. (E) After one cycle of V.A.C. VERAFLO™ Therapy for four days.

A 41-year-old male, known case of complicated type 2 DM and left diabetic foot with big toe amputation, was admitted on 23/7/2018 with wet gangrene of the left second toe and infected forefoot. His blood workup showed severe leukocytosis of 42 x 10^9^/L and mild renal impairment with uncontrolled blood sugar of 19 mmol/L. The patient was started on intravenous (IV) antibiotics and insulin infusion and underwent surgical debridement and left second toe amputation. He needed three more surgical debridement followed by amputation of left third toe (on 2/8/2018, 12/8/2018 and 20/8/2018). Six days after last debridement (Figure 1A) decision was made to fix CLEANSE CHOICE™ Dressing with V.A.C. VERAFLO™ Therapy. We used MicroSafe® (Sonoma Pharmaceuticals, Petaluma, CA) as instillation fluid, 20 cc with soak time of 15 minutes every four hours with V.A.C pressure of 75 mm Hg. Three days later, the wound bed showed dramatic improvement (Figure 1B), so a second application of the CLEANSE CHOICE™ dressing for another three days was done with reducing the frequency of instillation to six hourly. Figure 1C showed the wound bed of the second application and Figure 1D showed necrotic slough attached to the sponge of CLEANSE CHOICE™. Since the remaining necrotic and infected tissue was significantly less, we used the usual foam dressing for V.A.C. VERAFLO™ Therapy for four days. Figure 1E is the end result. So, in total of nine days we were able to clean the wound bed and produce clean and healthy granulation without taking the patient to OR since he already had four times OR visits. The plan is to obtain wound closure by secondary intention healing.

Case 2 - abdominal wall

**Figure 2:**
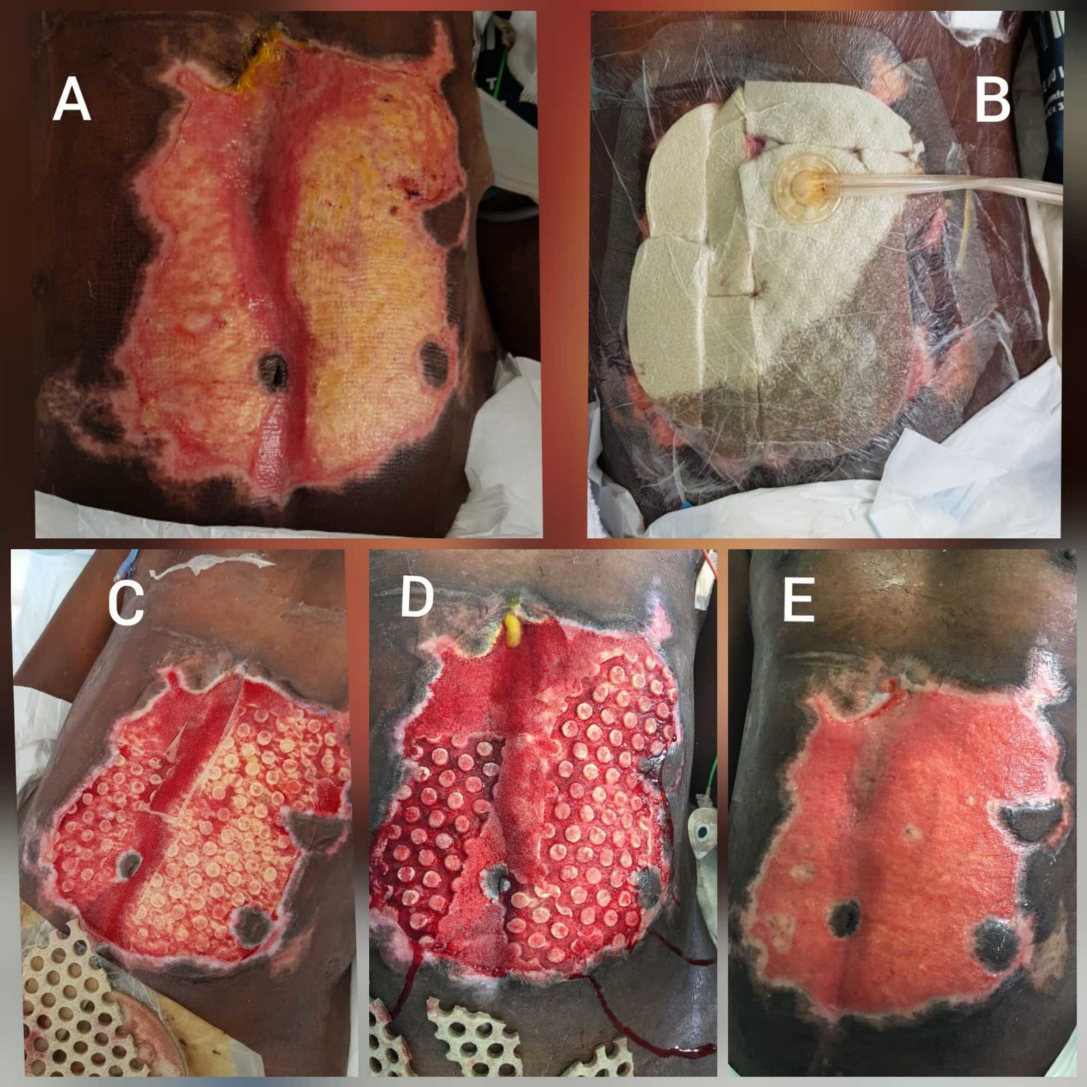
Case 2 (abdominal wall). (A) Abdominal wound before application. (B) V.A.C. VERAFLO CLEANSE CHOICE™ Dressing in situ. (C) Wound bed after first dressing. (D) Wound bed after second application. (E) After four days of simple VAC application.

A 31-year-old male, victim of road traffic accident, admitted to surgical intensive care unit (ICU) intubated with severe head injury in the form of multiple facial fractures and brain contusions. He had multiple bilateral flail rib fractures, lung contusions and pneumothorax for which bilateral chest tubes were fixed. He had five laparotomies during first 10 days for liver injury (segment IV B & V resection), hepatobiliary anastomosis for bile leak. We were consulted on hospital day 60 for a large full thickness abdominal skin loss with necrotic slough post-surgical debridement for gangrenous skin patch with methicillin-resistant Staphylococcus aureus (MRSA) and Acinetobacter (Figure 2A). He was a poor surgical candidate, so a decision was made to place V.A.C. VERAFLO CLEANSE CHOICE™ Dressing (Figure 2B). The settings were 50 cc of MicroSafe solution with a 20-minute dwell time, followed by six hours of NPWT at -100 mm Hg. After four days, half of necrotic slough was removed (Figure 2C) and almost 90% after the second application (Figure 2D). New cultures were collected and appeared negative. The wound was then fixed to the simple V.A.C for another four days. The final wound was ready for skin grafting but because the patient was poor surgical candidate and developed biliary fistula, decision was taken to leave the wound to heal by secondary intention (Figure 2E).

Case 3 - dehiscence of amputation stump

**Figure 3:**
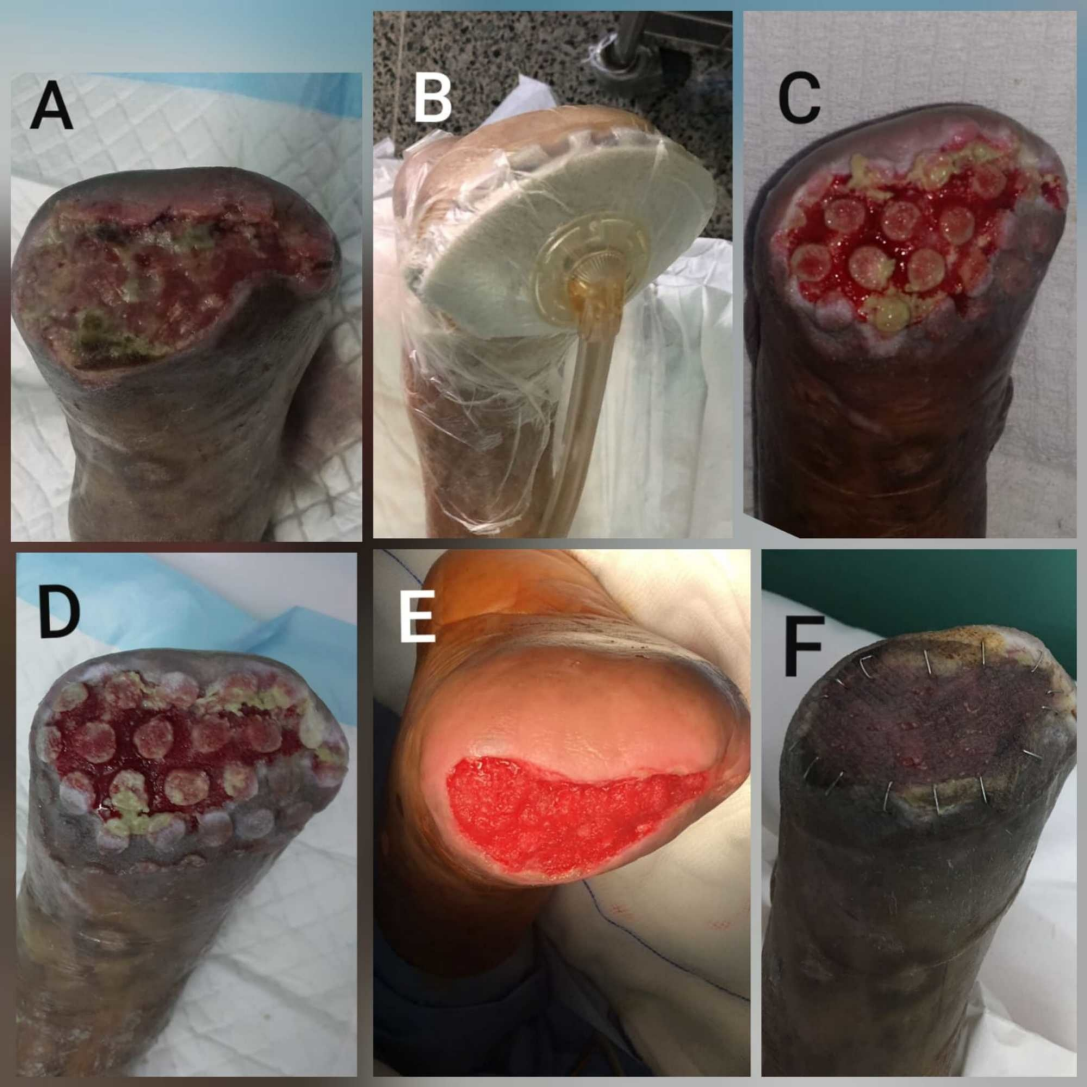
Case 3 (dehiscence of amputation stump). (A) Amputation stump wound prior to application. (B) V.A.C. VERAFLO CLEANSE CHOICE™ Dressing was applied. (C) After four days, more than 50% necrotic slough was removed. (D) Almost all necrotic slough was removed after the second application. (E) After four days of V.A.C. VERAFLO™ therapy, the wound was ready for grafting. (F) Skin graft with 100% take.

A 63-year-old man, known case of DM type II, hypertension and peripheral vascular disease, developed right forefoot dry gangrene. He underwent right femoroperoneal bypass and the gangrenous forefoot was treated conservatively. Six months later, he was admitted with wet gangrene of the forefoot for which he underwent right trans-metatarsal amputation. He developed wound dehiscence and gangrene of the skin of the amputation stump, for which he underwent surgical debridement. We were consulted for wound coverage; the wound bed was not yet ready. Figure 3A shows the necrotic bed with ischemic skin edges. V.A.C.VERAFLO CLEANSE CHOICE™ Dressing was fixed with installation of 15 cc of MicroSafe® and soak time of 15 minutes every six hours with V.A.C pressure of 75 mm Hg (Figure 3B). After four days, more than 50% necrotic slough was removed (Figure 3C) and almost all after the second application (Figure 3D). He was transitioned to V.A.C. VERAFLO™ without the cleanse dressing and after two applications was ready for grafting (Figure 3E), and the skin graft take was 100% (Figure 3F). The patient was able to be discharged with covered and stable amputation stump in two weeks with only single visit to OR.

## Discussion

Negative pressure wound therapy with instillation (NPWTi) and dwell time is an adjunctive treatment modality for selected complex chronic wounds. The recommended dwell time can range from 10 to 20 minutes, and the negative pressure time may vary from two to four hours at a pressure of -125 mm Hg, although time of up to six hours may be needed for larger wounds [3]. To avoid impairment of limb perfusion, we use lower negative pressure in lower limbs of diabetic and/or ischemic patients, usually -75 mm Hg. The type of instillation fluid used in NPWTi-d has been repeatedly studied and showed no difference between normal saline, Dakin’s solution, Microcyn, chlorhexidine or diluted Betadine. Instillation of normal saline can achieve comparable outcomes to other types of solution. Selecting an instillation solution should be largely based on its tolerability, spectrum of activity, availability, and cost [3]. We routinely use microsafe (Microcyn®) [8] as our instillation fluid in all our cases of NPWTi-d because it is extensively available in our gulf area.

V.A.C. VERAFLO CLEANSE CHOICE™ Dressing is a novel, adjunctive non-surgical option that may help clean large complex wounds when complete surgical debridement is not possible or appropriate. Prior to our paper, only eight articles using this type of dressing have been published, all are case reports of one or two cases except one [9-11]. In the first published peer-reviewed paper by Téot et al., they showed reduction in the surface area of black non-viable tissue and yellow fibrinous slough to less than 10% in 18 out of 21 patients (85.7%) and 15 out of 21 (71.4%) wounds had ≤20% surface area with yellow fibrinous slough remaining. Of 21 wounds, 20 (95.2%) displayed enhanced granulation tissue formation and reduction in wound volume [9]. In all of our three cases, all the wounds had less than 5% remaining surface area with yellow slough.

Investigators observed that greater than nine days of therapy appeared to result in enhanced growth of tissue into the dressing and pain at dressing changes. A decision was made to discontinue the dressings after three dressing changes [9]. In all our cases we stopped the new dressing after two dressing changes, replaced by one dressing of NPWTi-d. In most published case reports, the dressing was used safely in severely debilitated as well as frail patients [10]. We found the same in our cases, the use was safe with no harm or complication related to the foam dressing application or removal. We routinely soak the dressing few minutes before removal.

To date, limited data is available regarding the exact mechanism of action of the Cleanse Choice foam dressing. Expert panel of clinicians postulated recommendations and algorithm that can serve as a guide for clinicians until more studies are published [11]. They suggest the following goals for using V.A.C. VERAFLO CLEANSE CHOICE™ Dressing: (1) cleanse wounds when areas of slough or nonviable tissue remain present on the wound surface, (2) remove thick exudate, (3) remove infectious materials, (4) promote granulation tissue formation, and (5) help provide a bridge to a defined endpoint for a clinical plan of care [11].

Although surgical debridement is faster and more effective, it always carries the risk of over-excision and damage to healthy parts of the wound. This in turn will delay wound healing. In addition to the high cost of visiting operation room, anesthesia for medically high-risk patients is not always feasible or appropriate. Thus using V.A.C.VERAFLO CLEANSE CHOICE™ Dressing together with NPWTi-d helps removing wound bioburden at the bedside, which could further help facilitate wound healing at a reduced cost compared with repeated visits to operation room to undergo repeated surgical debridement.

## Conclusions

We found that using V.A.C. VERAFLO CLEANSE CHOICE™ Dressing with the V.A.C. ULTA™ Therapy System helped loosen, detach and removal of viscous wound exudate, fibrinous slough and infectious materials from the wound bed. This novel technique may be considered when surgical debridement is not possible or not appropriate. However, large, controlled clinical studies are needed to determine the optimal therapy settings and greatest effectiveness for the use of NPWTi-d with V.A.C. VERAFLO CLEANSE CHOICE™ Dressing.
